# The Interaction of Adrenomedullin and Macrophages Induces Ovarian Cancer Cell Migration via Activation of RhoA Signaling Pathway

**DOI:** 10.3390/ijms14022774

**Published:** 2013-01-29

**Authors:** Xiaoyan Pang, Hai Shang, Boya Deng, Fang Wen, Yi Zhang

**Affiliations:** 1 Department of Gynecology, The First Affiliated Hospital of China Medical University, Shenyang 110001, Liaoning, China; E-Mails: pxy-2011@hotmail.com (X.P.); frozenroses@yahoo.cn (B.D.); 2 Department of Hepatobiliary Surgery, Liaoning Tumor Hospital, Shenyang 110042, Liaoning, China; E-Mail: fc325545@sina.com

**Keywords:** ovarian cancer, adrenomedullin, macrophages, migration, RhoA

## Abstract

Tumor-associated macrophages (TAMs) are correlated with poor prognosis in many human cancers; however, the mechanism by which TAMs facilitate ovarian cancer cell migration and invasion remains unknown. This study was aimed to examine the function of adrenomedullin (ADM) in macrophage polarization and their further effects on the migration of ovarian cancer cells. Exogenous ADM antagonist and small interfering RNA (siRNA) specific for ADM expression were treated to macrophages and EOC cell line HO8910, respectively. Then macrophages were cocultured with HO8910 cells without direct contact. Flow cytometry, Western blot and real-time PCR were used to detect macrophage phenotype and cytokine production. The migration ability and cytoskeleton rearrangement of ovarian cancer cells were determined by Transwell migration assay and phalloidin staining. Western blot was performed to evaluate the activity status of signaling molecules in the process of ovarian cancer cell migration. The results showed that ADM induced macrophage phenotype and cytokine production similar to TAMs. Macrophages polarized by ADM promoted the migration and cytoskeleton rearrangement of HO8910 cells. The expression of RhoA and its downstream effector, cofilin, were upregulated in macrophage-induced migration of HO8910 cells. In conclusion, ADM could polarize macrophages similar to TAMs, and then polarized macrophages promote the migration of ovarian cancer cells via activation of RhoA signaling pathway *in vitro*.

## 1. Introduction

Epithelial ovarian cancer (EOC) is the most leading cause of death in women with gynecologic malignancies worldwide [1]. Most patients are diagnosed at an advanced stage due to occult onset and easily metastasis. However, the mechanism underlying the aggressiveness of EOC remains largely unclear. Accumulating evidences suggest that the progression of EOC is highly influenced by both the host immune response and inflammatory cells within the tumor microenvironment. Among inflammatory cells, macrophages are believed to play a pivotal role.

Macrophages have functional plasticity and can change their functional profiles repeatedly in response to environmental changes [2]. When exposed to lipopolysaccharides (LPS) and interferon-γ (IFN-γ), they are polarized to classical activated macrophages (M1) and have antitumor activities. In stark contrast, when encountering factors, like IL-4, IL-10 and IL-13, they are polarized to alternatively activated macrophages (M2) and possess pro-tumor abilities [3–5]. Compared with M1, M2 macrophages produce low amounts of inducible NOS (iNOS), tumor necrosis factor-α (TNF-α) and IL-1, but a higher amount of arginase 1 (Arg-1), IL-10 and express M2-specific surface markers, such as CD206 [6]. Tumor-associated macrophages (TAMs), referring to the macrophages residing in the tumor microenvironment, are polarized M2 macrophages. TAMs can promote tumor growth, angiogenesis, invasion and metastasis.

Adrenomedullin (ADM) is a potent vasodilator peptide originally isolated from human pheochromocytoma [7]. ADM expression was observed in various kinds of tumors, including breast, lung, colon and ovarian cancer [8]. It is a multifunctional regulatory peptide that is involved in angiogenesis, cell proliferation, apoptosis survival and immune escape [9]. Our previous study demonstrated that ADM contributed to the progression of EOC [10]. Thus, we supposed that ADM might play an important role in EOC invasion and metastasis. Subsequently, a recent study reported that TAMs enhanced angiogenesis and melanoma growth via ADM [11]. However, the relationship between TAMs and ADM in EOC has not been determined.

In the present study, we found that tumor-derived ADM could polarize macrophages similar to TAMs, and then polarized macrophages promote the migration of ovarian cancer cells via activation of RhoA signaling pathway *in vitro*.

## 2. Results and Discussion

### 2.1. Mice Peritoneal Macrophages Isolated and Determined

**Figure 1:**
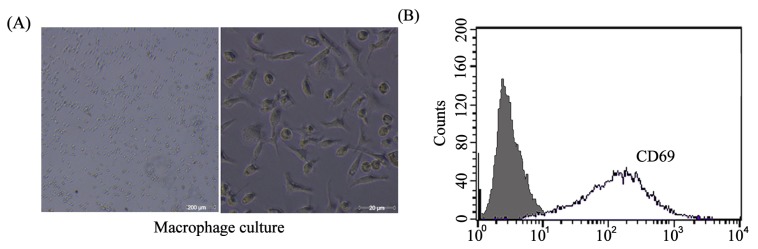
Mice peritoneal macrophage culture and determination of surface marker. (**A**) Mice peritoneal macrophages were isolated and cultured. Original magnification is 100× (left) or 400× (right); (**B**) Flow cytometry showed a significant induction for CD 69 in macrophages.

Mice were sacrificed, and peritoneal macrophages were isolated and cultured (Figure 1A). Flow cytometry was used to determine the surface marker of the macrophages. As shown in Figure 1B, macrophages showed a significant induction for CD 69 (marker for macrophages differentiation).

### 2.2. ADM Induced Macrophage Phenotype and Cytokine Production Similar to TAMs

**Figure 2:**
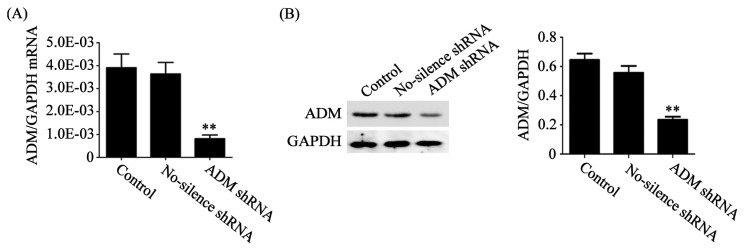
Knockdown of adrenomedullin (ADM) expression in HO8910 cells with specific shRNA. (**A**) The mRNA level of ADM was quantified by real-time Polymerase Chain Reaction (PCR); (**B**) The protein level of ADM was measured by Western blot. The relative expression of ADM was normalized to glyceraldehyde 3-phosphate dehydrogenase (GAPDH). Data are presented as the mean ± SD of triplicate experiments. ** *p* < 0.01 *vs*. control.

**Figure 3:**
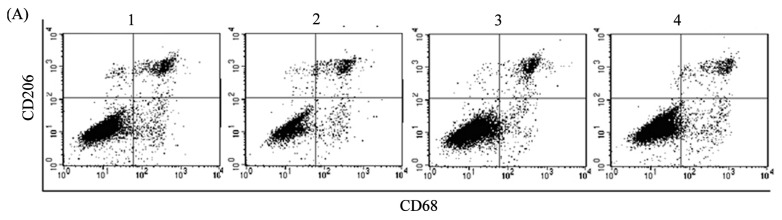

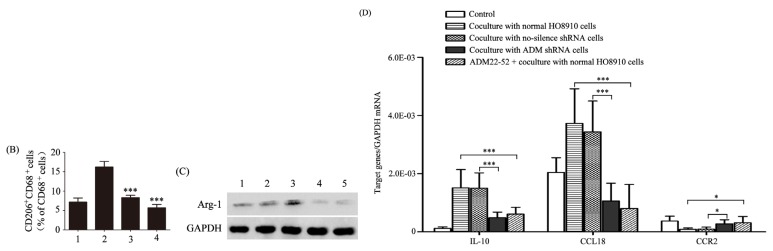
ADM induced macrophage phenotype and cytokine production similar to tumor-associated macrophages (TAMs). Macrophages were treated with or without ADM22-52, the antagonist of ADM (1 nM) for 1 h, then cocultured with normal or ADM knockdown HO8910 cells for 24 h. (**A**) Macrophages were cocultured with normal HO8910 cells for 24 h in the presence of ADM22-52 or knockdown of ADM, and the expression of CD206 was assessed by flow cytometry; (**B**) The expression of CD206 was significantly decreased in the presence of ADM22-52 or knockdown of ADM in HO8910 cells. Data are presented as the mean ± SD of triplicate experiments. *** *p* < 0.001 *vs*. coculture with normal HO8910 cells. Panels: 1, control; 2, coculture with normal HO8910 cells; 3, coculture with ADM shRNA cells; 4, pretreatment with ADM22-52 and then coculture with normal HO8910 cells; (**C**) Macrophages were cocultured with normal HO8910 cells for 24 h in the presence of ADM22-52 or knockdown of ADM, and the expression of Arg-1 was analyzed using Western blot. Lanes: 1, control; 2, coculture with normal HO8910 cells; 3, coculture with no-silence shRNA cells; 4, coculture with ADM shRNA cells; 5, pretreatment with ADM22-52 and then coculture with normal HO8910 cells; (**D**) The mRNA levels of IL-10, CCL18 and CCR2 in cocultured macrophages were examined by real-time PCR in the presence of ADM22-52 or knockdown of ADM in HO8910 cells. The relative expression levels of target genes were normalized to GAPDH. Data are presented as the mean ± SD of triplicate experiments. * *p* < 0.05, *** *p* < 0.001 between indicated pairs.

To examine the effect of tumor-derived ADM on macrophage polarization, we constructed a stable HO8910 cell line with ADM knockdown (Figure 2) or pretreated macrophages with ADM22-52, the antagonist of ADM, to block the function of ADM in macrophages. We then used macrophages pretreated with or without ADM22-52 to coculture with normal or ADM knockdown HO8910 cells for 24 h in a noncontact system. After coculture, the macrophages were harvested and assayed for phenotype and cytokine production. High expression of CD206 reflects the tendency of macrophage skewing toward M2 phenotype [6]. CD68 was expressed on both M1 and M2 macrophages. We found that the expression of CD206 was significantly increased in macrophages after coculture with normal HO8910 cells compared with the control, whereas a noticeable reduction of CD206 expression was detected in the presence of ADM22-52 or knockdown of ADM in HO8910 cells (Figure 3A,B). To explore the secretion effect of ADM on macrophages, Arg-1, the marker for M2 macrophages, was also examined in macrophages after coculture with normal HO8910 cells. The results showed that the expression of Arg-1 was greatly increased compared with the control, and the increments were abrogated in the presence of ADM22-52 or knockdown of ADM in HO8910 cells (Figure 3C). To further demonstrate macrophage polarization, real-time PCR was used to detect cytokines and chemokines in macrophages after coculture with normal HO8910 cells (Figure 3D). We found that the mRNA levels of IL-10 and CCL18 were obviously increased compared with the control, whereas ADM22-52 or knockdown of ADM in HO8910 cells attenuated the effects. We also observed lower mRNA level of CCR2 compared with the control, which was significant increased in the presence of ADM22-52 or knockdown of ADM in HO8910 cells. These results clearly supported that tumor-derived ADM could induce macrophage phenotype and cytokine production similar to TAMs.

### 2.3. Macrophages Polarized by ADM Promoted the HO8910 Cell Migration

**Figure 4:**
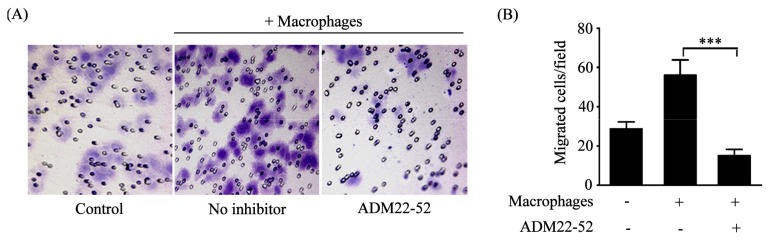

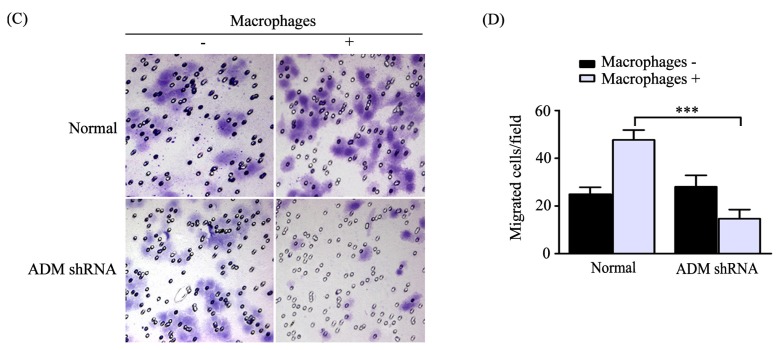
Macrophages polarized by ADM promoted the HO8910 cell migration. (**A**) Macrophages were pretreated with ADM22-52 (1 nM) for 1 h and then cocultured with normal HO8910 cells for 24 h. The migration ability of HO8910 cells was assessed by Transwell migration assay; (**B**) The number of migrated cells in cocultured HO8910 cells was greatly reduced in the presence of ADM22-52. Data are presented as the mean ± SD of triplicate experiments. ***p < 0.001 between indicated pair; (**C**) ADM knockdown HO8910 cells were cocultured with macrophages for 24 h, and then the migration ability was examined; (**D**) ADM knockdown significantly decreased the number of migrated cells in cocultured HO8910 cells. Data are presented as the mean ± SD of triplicate experiments. *** *p* < 0.001 between indicated pair.

To investigate the effect of macrophages polarized by ADM on ovarian cancer cells, we first assessed the migration ability of normal HO8910 cells after coculture with macrophages, as described in the Methods. The results showed that the migrated cells were obviously increased compared with the control, whereas pretreatment macrophages with ADM22-52 attenuated the effect (Figure 4A,B). We also found that after coculture with macrophages, ADM knockdown led to significantly decreased migrated cells compared with normal HO8910 cells, whereas knockdown of ADM in HO8910 cells only showed no effect compared with normal HO8910 cells (Figure 4C,D). Thus, these results indicated that polarized macrophages promoted the HO8910 cell migration.

### 2.4. Macrophages Polarized by ADM Induced HO8910 Cell Cytoskeleton Rearrangement

**Figure 5:**
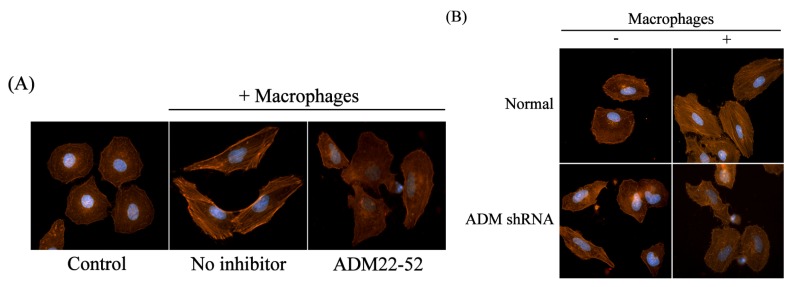
Macrophages polarized by ADM induced HO8910 cell cytoskeleton rearrangement. (**A**) Macrophages were pretreated with ADM22-52 (1 nM) for 1 h and then cocultured with normal HO8910 cells for 24 h. The stress fiber formation and cytoskeleton rearrangement of HO8910 cells were significantly reduced in the presence of ADM22-52, detected by phalloidin staining; (**B**) ADM knockdown cells were cocultured with macrophages for 24 h, and then the stress fiber formation and cytoskeleton rearrangement were examined using phalloidin staining. ADM knockdown obviously decreased the stress fiber formation and cytoskeleton rearrangement of cocultured HO8910 cells.

Cell migration is driven by the mechanical force provided by dynamic remodeling of the actin cytoskeleton [12]. Consequently, we examined the stress fiber formation and cytoskeleton rearrangement in normal cocultured HO8910 cells using phalloidin staining. The results showed that stress fiber formation and cytoskeleton rearrangement were significantly increased compared with the control, whereas this alteration was abrogated in the presence of ADM22-52 (Figure 5A). Furthermore, after coculture with macrophages, downregulation of ADM led to an obviously decreased stress fiber formation and cytoskeleton rearrangement compared with normal HO8910 cells, whereas knockdown of ADM in HO8910 cells only had no effect compared with normal HO8910 cells (Figure 5B). These results suggested that polarized macrophages induced HO8910 cell cytoskeleton rearrangement.

### 2.5. Macrophage-Induced Migration of HO8910 Cells via Activation of RhoA Signaling Pathway

**Figure 6:**
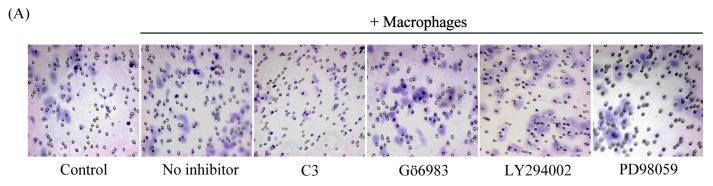

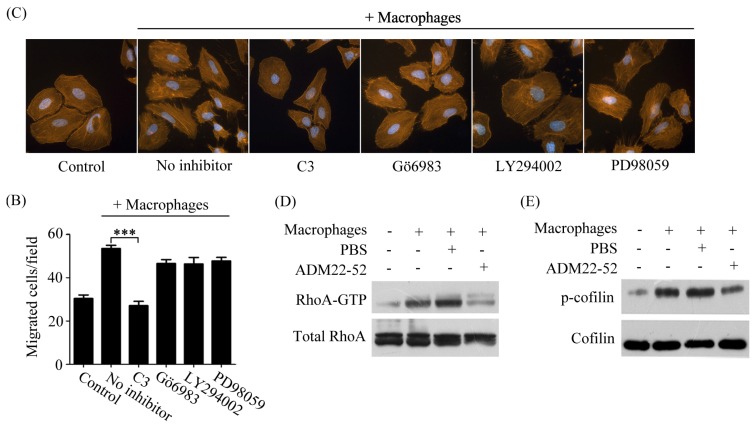
Macrophage-induced migration of HO8910 cells via activation of RhoA signaling pathway. (**A**) HO8910 cells were pretreated with C3 (5 μM), Gö6983 (100 nM), LY294002 (25 μM) and PD98059 (10 μM) for 1 h and then cocultured with macrophages for 24 h. The migration ability of HO8910 cells was examined by Transwell migration assay; (**B**) Pretreatment with C3 significantly reduced the migration ability of HO8910 cells. Data are presented as the mean ± SD of triplicate experiments. *** *p* < 0.001 *vs*. no inhibitor; (**C**) HO8910 cells were pretreated with inhibitors above for 1 h and then cocultured with macrophages for 24 h. The stress fiber formation and cytoskeleton rearrangement were detected by phalloidin staining; (**D**) Macrophages were pretreated with ADM22-52 (1 nM) for 1 h and then cocultured with normal HO8910 cells for 24 h. GTP-binding RhoA was detected by RhoA activity assay, followed by Western blot; (**E**) Macrophages were pretreated with ADM22-52 (1 nM) for 1 h and then cocultured with normal HO8910 cells for 24 h. Phosphorylated cofilin was accessed by Western blot.

Strong evidence indicated that cancer cell migration was regulated by several components of the intracellular signaling pathways, including RhoA, protein kinase C (PKC), phosphoinositide 3-kinase (PI3K) and extracellular regulated protein kinase (ERK) [13–16]. To define the intracellular effectors that were responsible for macrophage-induced migration of cancer cells, HO8910 cells were pretreated with the specific inhibitors for the signaling molecules mentioned above. We noticed that only pretreatment with C3 exoenzyme significantly reduced the migration ability of HO8910 cells (Figure 6A,B). In contrast, Gö6983, LY294002 and PD98059 had no such effect. Moreover, the stress fiber formation and cytoskeleton rearrangement of HO8910 cells were consistently greatly inhibited by pretreatment of C3 exoenzyme, whereas other inhibitors showed no such effect (Figure 6C). It is well known that C3 exoenzyme is the specific inhibitor of three isoforms of Rho, RhoA, RhoB and RhoC. These results demonstrated that Rho was involved in macrophage-induced migration of HO8910 cells.

Rho is a molecular switch that depends on the cycling of GDP and GTP-bound form. In its GTP-bound form, Rho activates downstream kinases, ROCK and LIMK, in turn. Activated LIMK can inactivate cofilin by inducing phosphorylation of cofilin, while non-phosphorylated cofilin can induce actin depolymerization [17–19]. To further confirm the inhibition experiments, we detected the activity status of signaling molecules in HO8910 cells after coculture with macrophages. Here, we employed anti-RhoA antibody to examine the RhoA activation. The results showed that GTP-binding RhoA was significantly increased, while the increment was abrogated in the presence of ADM22-52 (Figure 6D). We also observed a similar effect in phosphorylated cofilin (Figure 6E). Taken together, these results suggested that a signaling cascade downstream of RhoA was involved in macrophage-induced migration of HO8910 cells.

### 2.6. Discussion

It is now widely accepted that TAMs play a pivotal role in regulating tumor invasion and metastasis. Clinical studies showed that extensive TAM infiltration was associated with disease progression and poor prognosis in patients with breast, cervix and prostate cancer [20], yet, the precise mechanism by, which TAMs facilitate ovarian cancer cells migration and invasion remains unknown. In this study, we used a noncontact system described previously for coculture macrophages and ovarian cancer cells. The result showed that ADM, derived from ovarian cancer cells, could polarize macrophages similar to TAMs, which in turn promote the migration of ovarian cancer cells via activation of the RhoA signaling pathway.

Our previous study demonstrated that ADM is a factor of biological aggressiveness in EOC patients [10], which was in agreement with the result shown by Hata K. *et al*. [21]. However, a recent study reported the opposite, namely that high expression level of ADM in primary tumors was related to a better outcome. They also observed that, for the vasoactive properties, actually the peptide could increase perfusion and chemotherapy sensitivity [22]. The inconsistency might be attributed to the differences within the study populations and/or designs, which makes the precise function of ADM in this disease more complicated. Accumulating studies suggest that macrophages in the tumor microenvironment can be polarized to M2 phenotype [3], whereas the role of ADM in this process remains poorly clarified. A recent study showed that TAM-derived ADM contributed to macrophage polarization in an autocrine manner to promote angiogenesis and melanoma growth [11]. Interestingly, our results revealed that ADM, derived from ovarian cancer cells, correlated with macrophage polarization *in vitro*. Our present study showed that ovarian cancer cells switched phenotype and cytokine production of cocultured macrophages similar to TAMs, which exhibited increased expression levels of CD206, Arg-1, IL-10 and CCL18 and a lower expression level of CCR2. It is known that CCL18, the most abundant chemokine in human ovarian ascites, was of M2 origin [23]. IL-10 is produced by a variety of tumor cells, and TAMs and accounts for CCL18 production by TAMs [3]. Downregulation of CCR2 is somewhat consistent with a recent study, showing that TAMs isolated from various murine tumors and from human ovarian cancer express low levels of CCR2 in particular [24]. Our further data revealed that macrophage polarization was attenuated by ADM antagonist or knockdown of ADM in ovarian cancer cells. Consistently, Baranello C. *et al*. noticed *in vitro* the capability of ADM to stimulate M2 differentiation [22]. We therefore considered that ADM, derived from ovarian cancer cells, might be a novel target to inhibit macrophages polarization.

Using *in vitro* Transwell migration assay, we demonstrated that polarized macrophages could promote ovarian cancer cell migration. Further results showed that the migration ability of cocultured ovarian cancer cells was effectively blocked in the presence of ADM antagonist or knockdown of ADM in ovarian cancer cells, which was different from our previous studies showing that ADM had an autocrine effect on ovarian cancer cells [10]. Interestingly, downregulation of ADM only showed a similar migration effect compared with normal ovarian cancer cells, which excluded the possible functions of endogenous ADM in ovarian cancer cells. In addition, our results confirmed that ADM antagonist or knockdown of ADM in ovarian cancer cells could reverse the effect that polarized macrophages induced cytoskeleton rearrangement of ovarian cancer cells. Collectively, these observations suggested that ADM, derived from ovarian cancer cells, was a key factor for macrophage-induced migration and cytoskeleton rearrangement of ovarian cancer cells. ADM, therefore, represented an important link between TAMs and ovarian cancer cell migration.

Polarized macrophages may activate several signaling pathways to promote cancer cell migration. In this study, we found that RhoA signaling was activated in macrophage-induced migration of ovarian cancer cells. Based on the observed results, we suppose that the interaction between ADM and macrophages might be a signal for RhoA activation, which correlates with the subsequent events, including phosphorylation of cofilin, cytoskeleton rearrangement and ovarian cancer cell migration.

## 3. Materials and Methods

### 3.1. Cell Culture

Human EOC cell line HO8910 cells were maintained in RPMI 1640 medium supplemented with 10% fetal bovine serum (Invitrogen, Carlsbad, CA, USA) and cultured at 37 °C with 5% CO_2_.

### 3.2. Knockdown of ADM Expression with Specific shRNA

The ADM-specific short hairpin RNA (shRNA) and no-science shRNA were designed and constructed in our previously study [25]. The oligonucleotide shRNAs based on the small interfering RNA (siRNA) sequences were cloned into pRNA-U6.1/Neo (GeneScript, Piscataway, NJ, USA), respectively. The constructs were transfected into HO8910 cells using Lipofectamine 2000 (Invitrogen, Carlsbad, CA, USA) and selected by G418 (Invitrogen, Carlsbad, CA, USA). Stable cell clones were identified by Western blot.

### 3.3. Western Blot

The cells were lysed in RIPA buffer (50 mM Tris-HCL, 150 mM NaCl, 1% NP-40, 0.5% deoxycholate, 0.1% sodium dodecylsulfate) supplemented with protease inhibitors. Protein concentrations were determined using BCA protein assay reagent kit (Pierce Chemical Co., Rockford, IL, USA). Equal amounts of protein were separated by SDS-PAGE and transferred to PVDF membrane followed by immunoblotting. Membranes were then incubated overnight at 4 °C with primary antibodies for ADM (1:800, Sigma, St. Louis, MO, USA), Arg-1 (1:1000, Santa Cruz Biotechnology, Santa Cruz, CA, USA), p-cofilin (1:1000, Cell Signaling Technology Inc., Danvers, MA, USA), cofilin (1:1000, Cell Signaling Technology Inc., Danvers, MA, USA) and GAPDH (1:1000, Santa Cruz Biotechnology, Santa Cruz, CA, USA) and subsequently incubated with horseradish peroxidase-conjugated secondary antibodies for 1 h at room temperature. Protein bands were visualized using Amersham ECL plus Western Blotting Detection Reagents (GE Healthcare, Diegem, Belgium). Quantification of band density was done using Alpha Ease Fc software (Alpha Innotech, CA, USA).

### 3.4. Preparation of Peritoneal Macrophages and Culture

BALB/c female nude mice (6 weeks old, 16.0 ± 2.0 g) were purchased from Institute of Laboratory Animal Science, Chinese Academy of Medical Sciences (Beijing, China). Animal experiment was approved by the China Medical University Animal Care and Use Committee. Mice were sacrificed and peritoneal macrophages were isolated by peritoneal lavage with 3 mL RPMI 1640 without fetal bovine serum. The cells were centrifuged at 1000× *g* for 5 min, resuspended in RPMI 1640 containing 10% fetal bovine serum. The cells were seeded into 6-well plates at a concentration of 2 × 10^5^ cells/well. Following incubation in a humidified incubator for 24 h at 37 °C with 5% CO_2_, the cells were washed 3-times with culture medium before the specific treatment.

### 3.5. Macrophage and HO8910 Cell Coculture

Normal or ADM knockdown HO8910 cells were cocultured with macrophages without direct cell-to-cell contact, as described previously [26]. Briefly, 2 × 10^5^ macrophages were seeded into the upper chamber of Transwell (0.4 μm pore, Corning Costar Corp., Cambridge, MA, USA). Macrophages were pretreated with 1 nM ADM22-52, the ADM antagonist (Sigma, St. Louis, MO, USA), 1 h before coculture. After coculture for 24 h, the macrophages were harvested from the Transwell inserts for subsequent experiments.

### 3.6. Flow Cytometry

After blocking, cells were incubated with fluorescein isothiocyanate (FITC)-conjugated rat anti-mouse CD68 antibody (1:200, BD Biosciences, Franklin Lakes, NJ, USA), PE-conjugated rat anti-mouse CD206 antibody (1:200, BD Biosciences, Franklin Lakes, NJ, USA) and PE-conjugated rat anti-mouse CD69 (1:200, BD Biosciences, Franklin Lakes, NJ, USA) for 1 h at 4 °C. After washed twice by cold PBS, cells were analyzed by the FACSCalibur flow cytometry system (BD Biosciences, Franklin Lakes, NJ, USA).

### 3.7. Real-Time PCR

Total RNA was isolated with Trizol (Invitrogen, Carlsbad, CA, USA), and the reverse transcriptase reaction was carried out using a PrimeScript RT Reagent (Takara, Tokyo, Japan). Real-time PCR was performed with SYBR Premix Ex Taq Kit (Takara, Tokyo, Japan) on Applied Biosystems 7500 (Foster City, CA, USA). The sequences of primers and probes were listed in Table 1. Recombinant pGEM T-vectors (Promega, Madison, WI, USA) containing ADM, IL-10, CCL18, CCR2 or GAPDH cDNA were constructed and used to establish the standard curves.

### 3.8. Cell Migration Assay

The cell migration assay was conducted using 24-well Transwell inserts (8 μm pore, Corning Costar Corp., Cambridge, MA, USA). Briefly, 1 × 10^5^ normal or ADM knockdown HO8910 cells were seeded into the upper chamber of Transwell and 2 × 10^5^ macrophages were seeded into the lower chamber of Transwell. Macrophages were pretreated with 1 nM ADM22-52 1 h before coculture. After coculture for 24 h, cells on the upper surface of the insert membrane were removed with cotton rods, and the migrated cancer cells were fixed and stained with 0.05% crystal violet. The migrated cells were counted at 200× magnification in 10 different fields for each insert by microscopy.

For inhibitor experiments, HO8910 cells cultured in Transwell inserts were pretreated for 1 h with the inhibitors, including C3 (5 μM), Gö6983 (100 nM), LY294002 (25 μM) and PD98059 (10 μM) (Calbiochem, San Diego, CA, USA), respectively. Then, the culture medium was removed, and the inserts were loaded into the lower chamber of Transwell seeded with macrophages to examine the migration of cancer cells.

### 3.9. Cell Cytoskeleton Staining

Normal or ADM knockdown HO8910 cells were washed with PBS (pH 7.4) and fixed with 3.7% paraformaldehyde for 10 min. Subsequently, cells were washed with PBS and incubated with 0.1% Triton X-100 for 5 min. After blocking with 1% bovine serum albumin, cells were then incubated with FITC-phalloidin (Sigma, St. Louis, MO, USA) for 30 min. Images were captured at 1000× magnification by Laser confocal microscopy.

### 3.10. RhoA Activity Assay

The activation of RhoA in HO8910 cells cocultured with macrophages was detected by Rho Activation Assay Kit (Upstate Biotechnology, Lake Placid, NY, USA), according to the manufacturer’s instructions. Activated GTP-RhoA was detected by Western blot using rabbit anti-RhoA antibody (Santa Cruz Biotechnology, Santa Cruz, CA, USA).

### 3.11. Statistical Analysis

Data were analyzed with one-way ANOVA or Student *t* test using SPSS (version 16.0, Chicago, IL, USA). All the experiments were repeated at least three times. Data were presented as means ± standard deviation (SD). *p* < 0.05 was considered as statistically significant.

## 4. Conclusions

In summary, this study showed a novel connection among ADM, macrophages and ovarian cancer cell migration. Additional studies are needed to better define how these interactions are initiated and regulated and to demonstrate whether similar effects play a role *in vivo*.

## Figures and Tables

**Table 1:** The sequences of primers for real-time PCR.

Gene	Sequence
Homo-ADM	Forward: 5′-TCCCCCTATTTTAAGACGTGAATG-3′ Reverse: 5′-CATGCACACAAA CACACTCACAT-3′
Homo-GAPDH	Forward: 5′-GAAGGTGAAGGTCGGAGT-3′ Reverse: 5′-GAAAGATGGTGATGGGATTTC-3′
Mus-IL-10	Forward: 5′-TGAGGCGCTGTCGTCATCGATTTCTCCC-3′ Reverse: 5′-GGTTGCCAAGCCTTATCGGA-3′
Mus-CCL18	Forward: 5′-CCCTCCTTGTCCTCGTCTG-3′ Reverse: 5′-GCTTCAGGTCGCTGATGTATT-3′
Mus-CCR2	Forward: 5′-TTTGTTTTTGCAGATGATTCAA-3′ Reverse: 5′-TGCCATCATAAAGGAGCCAT-3′
Mus-GAPDH	Forward: 5′-TGCATCCTGCACCACCAACTGCTTAG-3′ Reverse: 5′-TTCACCACCATGGAGAAGGC-3′
